# Monitoring Intracellular Redox Changes in Ozone-Exposed Airway Epithelial Cells

**DOI:** 10.1289/ehp.1206039

**Published:** 2012-12-18

**Authors:** Eugene A. Gibbs-Flournoy, Steven O. Simmons, Philip A. Bromberg, Tobias P. Dick, James M. Samet

**Affiliations:** 1 Curriculum in Toxicology, University of North Carolina at Chapel Hill, Chapel Hill, North Carolina, USA; 2 Integrated Systems Toxicology Division, National Health and Environmental Effects Research Laboratory (NHEERL), U.S. Environmental Protection Agency (EPA), Research Triangle Park, North Carolina, USA; 3 Center for Environmental Medicine, Asthma and Lung Biology, University of North Carolina at Chapel Hill, Chapel Hill, North Carolina, USA; 4 Division of Redox Regulation, DKFZ-ZMBH Alliance, German Cancer Research Center (DKFZ), Heidelberg, Germany; 5 Environmental Public Health Division, NHEERL, U.S. EPA, Chapel Hill, North Carolina, USA

**Keywords:** glutathione, human airway epithelial cells, imaging, intracellular, oxidative stress, ozone, NADPH, redox, roGFP

## Abstract

Background: The toxicity of many xenobiotic compounds is believed to involve oxidative injury to cells. Direct assessment of mechanistic events involved in xenobiotic-induced oxidative stress is not easily achievable. Development of genetically encoded probes designed for monitoring intracellular redox changes represents a methodological advance with potential applications in toxicological studies.

Objective: We tested the utility of redox-sensitive green fluorescent protein (roGFP)–based redox sensors for monitoring real-time intracellular redox changes induced by xenobiotics in toxicological studies.

Methods: roGFP2, a reporter of the glutathione redox potential (*E*_GSH_), was used to monitor *E*_GSH_ in cultured human airway epithelial cells (BEAS-2B cells) undergoing exposure to 0.15–1.0 ppm ozone (O_3_). Cells were imaged in real time using a custom-built O_3_ exposure system coupled to a confocal microscope.

Results: O_3_ exposure induced a dose- and time-dependent increase of the cytosolic *E*_GSH_. Additional experiments confirmed that roGFP2 is not directly oxidized, but properly equilibrates with the glutathione redox couple: Inhibition of endogenous glutaredoxin 1 (Grx1) disrupted roGFP2 responses to O_3_, and a Grx1-roGFP2 fusion protein responded more rapidly to O_3_ exposure. Selenite-induced up-regulation of GPx (glutathione peroxidase) expression–enhanced roGFP2 responsiveness to O_3_, suggesting that (hydro)peroxides are intermediates linking O_3_ exposure to glutathione oxidation.

Conclusion: Exposure to O_3_ induces a profound increase in the cytosolic *E*_GSH_ of airway epithelial cells that is indicative of an oxidant-dependent impairment of glutathione redox homeostasis. These studies demonstrate the utility of using genetically encoded redox reporters in making reliable assessments of cells undergoing exposure to xenobiotics with strong oxidizing properties.

The intracellular redox environment is a highly dynamic setting governed by the formation and degradation of various reactive species of oxygen and nitrogen. Under normal physiological conditions, the cytosol, the nucleus, and the mitochondrial matrix space maintain homeostatic conditions in favor of a highly reducing environment (Cannon and Remington 2008). Intracellular reducing conditions are largely maintained by millimolar concentrations of reduced glutathione and its accessory enzymes that together constitute the glutathione system (Anderson 1998). Ultimately, maintenance of the intracellular glutathione redox potential (*E*_GSH_) comes from the metabolism of glucose because glutathione is reduced by glutathione reductase using NADPH (nicotinamide adenine dinucleotide phosphate) produced by the pentose phosphate pathway (PPP) (Wamelink et al. 2008).

A number of pathophysiological states are associated with changes in the *E*_GSH_ (Dubinina and Dadali 2010; Ma 2010; Yang and Omaye 2009). Such “oxidative stress” is commonly cited as a mechanistic feature of the toxicity of numerous xenobiotic compounds linked to adverse health outcomes (Bargagli et al. 2009; Ciencewicki et al. 2008; Kohen and Nyska 2002). For instance, the health effects of the potent ambient air pollutant ozone (O_3_) are understood to be mediated through an oxidative stress mechanism involving the oxidation of cellular biomolecules (Ballatori et al. 2009; Kelly et al. 1995; Mudway and Kelly 2000). In the lung, O_3_ exposure causes decrements in pulmonary function and induces inflammatory responses derived from the bronchial epithelium, a major target of O_3_ exposure (Ballinger et al. 2005; Ciencewicki et al. 2008; Kelly et al. 1995; Mudway and Kelly 2000; Pryor 1992; Pryor et al. 1995; Song et al. 2010). Because of its high reactivity, O_3_ interacts with cellular and extracellular biomolecules, resulting in multiple types of oxidative damage to lipids, proteins, and nucleic acids (Kelly et al. 1995; Laumbach 2010; Mudway and Kelly 2000; Srebot et al. 2009; van der Vliet et al. 1995; Yang and Omaye 2009). While numerous studies have established oxidant damage of biomolecules as a result of O_3_ exposure, direct measures of O_3_-mediated “oxidative stress” have been difficult to achieve; yet alteration of a defined intracellular redox couple like the glutathione redox pair (GSH/GSSG) would represent an important early indicator of the oxidative effects of O_3_ exposure.

Recent methodological advances have made it possible to focus studies of prooxidative changes to specific redox couples within defined subcellular compartments (Meyer and Dick 2010), potentially affording greater specificity in mechanistic investigations of the oxidative effects of xenobiotic exposures. A new generation of genetically encoded fluorophores permits direct assessment of the oxidative effects of xenobiotic compounds in relation to the GSH/GSSG redox pair with unprecedented spatial and temporal resolution (Cheng et al. 2012; Dooley et al. 2004; Meyer and Dick 2010). Redox-sensitive green fluorescent protein 2 (roGFP2) acts as a reporter of intracellular *E*_GSH_ by equilibrating with the GSH/GSSG redox pair (Gutscher et al. 2008; Meyer and Dick 2010; Morgan et al. 2011). In short, in a reaction that depends on catalysis by glutaredoxins, roGFP2 responds to oxidation of reduced glutathione (GSH) to its oxidized form (GSSG) via the internal formation of a disulfide bond (Gutscher et al. 2008; Meyer 2008; Meyer and Dick 2010) (Figure 1). The formation of the disulfide bond alters the spectral characteristics of the GFP fluorophore causing the intensity of the emitted green fluorescence (~ 520 nm) induced by excitation at 488 nm to decrease, while causing the emitted fluorescence after excitation at 405 nm to increase, thus making this sensor a ratiometric probe. Further efforts to improve the responsiveness of roGFP2 have led to the conjugation of pathway-specific enzymes to create a chimeric fusion of proteins operating as redox relays. In particular, the conjugation of glutaredoxin 1 (Grx1) to roGFP2 has been shown to enhance the kinetics of the roGFP2 response to the oxidation of glutathione (Gutscher et al. 2008).

**Figure 1:**
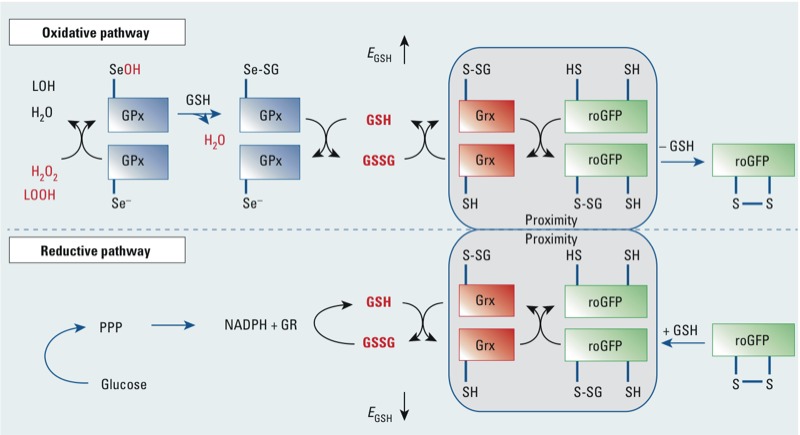
roGFP2 interactions with the glutathione system (adapted from Meyer and Dick 2010). Glutathione peroxidases (GPx) oxidize GSH to GSSG in response to peroxides, including H_2_O_2_ and lipid hydroperoxides (LOOHs), thus increasing the glutathione redox potential (*E*_GSH_). Abbreviations: LOH, reduced lipid oxide; Se^–^, reduced selenocyteine; SeOH, oxidized selenocyteine; SeSG, glutathionylated selenocysteine. In response to the increase in GSSG, one of the engineered vicinal cysteines of roGFP2 becomes *S*-glutathionylated by glutaredoxin (Grx). Glutathionylation in turn causes disulfide bond formation and alteration of the spectral properties of the GFP fluorophore. In the reductive pathway, Grx catalyzes the reduction of roGFP2 disulfide bonds through deglutathionylation as GSSG levels decrease and normal levels of GSH are reestablished by glutathione reductase (GR), at the expense of NADPH, causing a renormalization of *E*_GSH_. Glucose and the pentose-phosphate pathway (PPP) create NADPH, which is used by GR to reduce GSSG to GSH.

In the present study, we used live-cell microscopy to monitor the cytosolic *E*_GSH_ of airway epithelial cells undergoing exposure to O_3_ in real time. Here, we report an approach to validate the use of roGFP2-based redox sensors in toxicological studies of xenobiotics with strong oxidizing properties.

## Methods

*Materials and reagents*. We purchased tissue culture media and supplements from Lonza (Walkersville, MD, USA), and Wilco Wells glass-bottom culture dishes from Ted Pella Inc. (Redding, CA, USA) and Warner Instruments (Hamden, CT, USA). Fugene 6 transfection reagent was acquired from Roche Applied Science (Indianapolis, IN, USA). Kits to measure intracellular glutathione and NADPH were bought from Promega (Madison, WI, USA) and AbCam (Cambridge, MA, USA), respectively. Laboratory reagents and chemicals including hydrogen peroxide (H_2_O_2_), dithiothreitol (DTT), 2-acetylamino-3-[4-(2-acetylamino-2-carboxyethylsulfanylthiocarbonylamino)phenylthiocarbamoylsulfanyl]propionic acid (2-AAPA), buthionine sulfoximine (BSO), and sodium selenite were obtained from Sigma-Aldrich (St. Louis, MO, USA). Basic laboratory supplies were purchased from Fisher Scientific (Raleigh, NC, USA).

*Cell culture*. Transformed human airway epithelial cells [BEAS-2B, subclone S6 (Reddel et al. 1988)] were cultured as previously described (Tal et al. 2010) and maintained in serum-free keratinocyte growth medium (KGM; Lonza). The cells were incubated in a humidified incubator at 37°C in 5% carbon dioxide (CO_2_). For most live-cell exposures, BEAS-2B cells were plated in 35 mm Wilco Wells glass-bottom dishes with a 12 mm #1.5 glass aperture (Ted Pella Inc.).

*Genetically encoded redox sensors*. Plasmid for roGFP2 was the generous gift of S.J. Remington (University of Oregon, Eugene, OR, USA). Plasmid for the H_2_O_2_ sensor, HyPer, was purchased from Evrogen (Axxora, Farmingdale, NY, USA). Cytosolic and mitochondrially targeted versions of roGFP2 and HyPer were placed into lentiviral vectors as described previously (Cheng et al. 2012).

*Plasmid transfection and lentiviral transduction*. One to 2 days before exposure, BEAS-2B cells were transiently transfected with 1–2 µg of plasmid DNA encoding Grx1-roGFP2, HyPer, or roGFP2 using 3–5 µL of Fugene 6 transfection reagent for each 35 mm culture dish. Stable expression of a specific genetically encoded fluorescent reporter was performed via lentiviral transduction. In short, a lentivirus encoding roGFP2 or HyPer (i.e., specifically targeted to the cytosol or mitochondria, respectively) was incubated for 4 hr (at 37°C in 5% CO_2_) with wild-type BEAS-2B cells using a multiplicity of infection of 5–10 in a single well of a 6-well dish. The viral particles were then removed and fresh KGM was placed on the cells, and the BEAS-2B cells were then allowed to grow to confluency. Upon confluency, the cells were expanded to T75 dishes where they were propagated for multiple passages. For some experiments, stably transduced cells were sorted for optimal fluorescence expression at the University of North Carolina at Chapel Hill Core Flow Cytometry Facility.

*Exposure conditions*. Newly transfected or stably transduced BEAS-2B cells expressing the fluorescent reporter of interest were cultured as described above. Prior to exposure, subconfluent cells were equilibrated in Locke solution (Taylor-Clark and Undem 2010) for 2 hr at 37°C in 5% CO_2_. For the studies herein, two versions of Locke solution were derived by adding (LS + G) or excluding (LS – G) 1 mg/mL dl-glucose. Irrespective of the type of Locke solution used to equilibrate cells, all live-cell O_3_ exposures were performed using 0.5 mL of LS – G. For all imaging experiments, cells were exposed in a custom-built stage-top exposure system maintained at 37°C with 1.5 L/min of 5% CO_2_/balance air at a relative humidity of ≥ 95% [see Supplemental Material, Figure S1 (http://dx.doi.org/10.1289/ehp.1206039)]. In some experiments, cells were pretreated with 100 µM 2-AAPA, a dithiocarbamate inhibitor of glutaredoxins, during the 2-hr buffer equilibration period. Similarly, pretreatment of cells with 1 µM sodium selenite for 24–48 hr before O_3_ exposure was carried out to induce the overexpression of glutathione peroxidases (GPx).

For each experiment, cells were exposed to control air (5% CO_2_/balance air) or to O_3_ concentrations ranging from 0.15–1.0 ppm. The entire exposure period typically consisted of three component intervals collectively lasting ≤ 1 hr. They included *a*) an initial untreated baseline period of 5 min; *b*) an exposure period of ≤ 45 min; and *c*) a 10-min control exposure period in which cells were oxidized by 0.1–1.0 mM H_2_O_2_ for 5 min and then reduced by 10 mM DTT for an additional 5 min. During these exposures, the O_3_ concentration in the exposure chamber was monitored in real time using a Dasibi model 1003-AH O_3_ analyzer (Dasibi Environmental Corporation, Glendale, CA, USA) sampling at a flow of 2.0 L/min. O_3_ exposures for non-imaging assays were performed using exposure chambers operated by the U.S. Environmental Protection Agency’s Environmental Public Health Division.

*Imaging analysis*. All live-cell experiments done in real time were conducted using a Nikon Eclipse C1si spectral confocal imaging system equipped with an Eclipse Ti microscope, Perfect Focus System, and 404 nm, 488 nm, 561 nm, and 633 nm primary laser lines (Nikon Instruments Corporation, Melville, NY, USA). Images were acquired using a 60× Plan Apo lens. For experiments involving the genetically encoded fluorescent reporters, roGFP2 and HyPer, green fluorescence was observed via the use of independent excitations at 404 and 488 nm, and emitted light was collected for each using a 525/30 nm band-pass filter (Chroma, Bellows Falls, VT, USA). Results were calculated as ratios of the emissions excited by the 488 nm and 404 nm lasers scanned sequentially at a frequency of 1 min. All imaging data were acquired using Nikon EZ-C1 software.

*Measurement of intracellular NADPH*. After cells were equilibrated in fresh KGM or Locke solution (LS + G or LS – G) for 2 hr, the intracellular levels of total NADPH were assessed using an AbCam (Cambridge, MA, USA) NADP/NADPH assay kit according to the manufacturer’s instructions. Following the equilibration period, cells were immediately placed on ice and washed with cold 1× PBS just before the initial lysis step. Absorbance was read at 450 nm using a PolarStar Optima microplate reader (BMG Labtech, Durham, NC, USA).

*Statistical analysis*. All imaging data were quantified using NIS-Elements AR software (Nikon). For each experiment, the responses of 5–10 cells were collected as regions of interest and then averaged to derive an overall response. Data are expressed as the mean of at least three repeated experiments. Pairwise comparisons of control and treatment groups were performed using analysis of variance and linear regression, with *p* < 0.05 as statistically significant.

## Results

**Figure 2:**
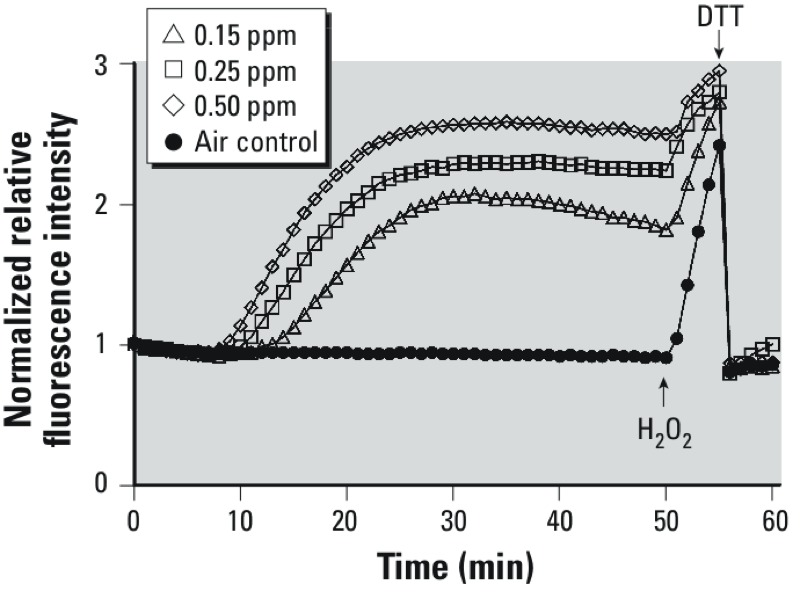
Exposure to O_3_ induces a dose- and time-dependent increase in the cytosolic *E*_GSH_ in airway epithelial cells. BEAS-2B cells expressing cytosolic roGFP2 were exposed to clean air for 5 min followed by a 0 (air control), 0.15, 0.25, or 0.50 ppm O_3_ exposure for 35 min in a stage-top exposure system maintained at 37°C, > 90% relative humidity, and 5% CO_2_. Shown are ratiometric values (404/488) calculated from the fluorescence intensity emitted at 510 nm induced by sequential excitation at 404 and 488 nm, plotted relative to the 5-min baseline. Addition of 0.1 mM H_2_O_2_ at the end of the O_3_ exposure produced a maximal response, which was fully reversible with the addition of 10 mM DTT. The data shown were derived from three or more separate experiments monitoring seven or more cells in real time throughout the exposure period.

*O_3_ exposure induces an increase in the cytosolic* E*_GSH_*. The presence of glucose in the exposure media is known to shorten the half-life of O_3_ (Taylor-Clark and Undem 2010). Therefore, in these experiments, cells were first equilibrated in LS + G for 2 hr, and then switched to LS – G for the exposure. Exposure of BEAS-2B cells expressing cytosolic roGFP2 to 0.15–0.50 ppm O_3_ resulted in a dose- and time-dependent probe response, reflecting an increase in the cytosolic *E*_GSH_ [Figure 2; see also Supplemental Material, Figure S5 (http://dx.doi.org/10.1289/ehp.1206039)]. Increasing O_3_ concentration hastened the onset while elevating the magnitude of the oxidative response reported by roGFP2 (Figure 2). Adding 0.1 mM H_2_O_2_ at the end of each O_3_ exposure produced a maximal response, which was fully reversible with the addition of 10 mM DTT.

**Figure 3:**
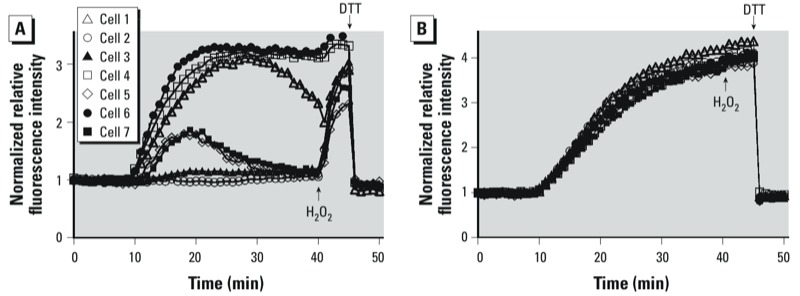
Glucose deprivation sensitizes cells to O_3_-induced roGFP2 oxidation. Shown are the responses of seven BEAS-2B cells equilibrated in Locke solution containing 1 mg/mL glucose (LS + G: *A*), or 0 mg/mL (LS – G *B*). Cells were exposed to 0.5 ppm O_3_; addition of 0.1 mM H_2_O_2_ at the end of the O_3_ exposure produced a maximal response, which was fully reversible with the addition of 10 mM DTT.

*Glucose deprivation potentiates the elevation of* E*_GSH_ induced by O_3_ exposure*. On an individual basis, BEAS-2B cells equilibrated in the presence of glucose (i.e., in LS + G) were observed to respond variably to a given concentration of O_3_, suggesting substantial heterogeneity of redox homeostasis within the cellular population under the given conditions. Figure 3A shows the individual responses of seven BEAS-2B cells in the same field of view being exposed to 0.5 ppm O_3_, with some cells responding with strongly increasing *E*_GSH_, while others responded only minimally. Furthermore, some cells exhibited an intermediate response followed by a recovery of *E*_GSH_ despite continued O_3_ exposure. Given the importance of PPP-generated NADPH in maintaining intracellular GSH levels, we hypothesized that glucose deprivation would sensitize the cells to a subsequent O_3_ exposure. As shown in Figure 3B, depriving the cells of glucose for 2 hr before exposure homogenized the magnitude, time of onset, and rate of response of the cells to O_3_. Glucose status did not affect the probe response to the addition of H_2_O_2_ and DTT (Figure 3A,B). As expected, glucose deprivation led to decreased cellular NADPH levels [see Supplemental Material, Figure S2 (http://dx.doi.org/10.1289/ehp.1206039)].

*Validation of glutathione-dependent roGFP2 responses to O_3_ exposure*. Given the extreme reactivity of O_3_ with biomolecules, we considered the possibility that the spectral changes of the probe interpreted as changes in *E*_GSH_ are the result of a direct oxidation of roGFP2 by O_3_ itself or by an O_3_-generated secondary oxidant. We therefore undertook a series of experiments to determine whether O_3_-induced changes in the roGFP2 fluorescence intensity ratio involve components of the glutathione system through which roGFP2 has been demonstrated to respond (Figure 1). We first aimed to confirm that O_3_ exposure leads to increased levels of GSSG. To this end we assessed the extent of intracellular glutathione oxidation in control and O_3_-treated cells. We observed ≤ 3-fold increases in GSSG following 1 ppm O_3_ exposure in LS – G as compared with air controls [see Supplemental Material, Figure S3 (http://dx.doi.org/10.1289/ehp.1206039)], which agrees with previous reports of the effect of O_3_ exposure on intracellular glutathione (Chalfant and Bernd 2011; Todokoro et al. 2004).

**Figure 4:**
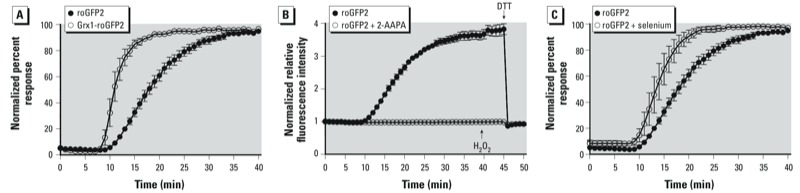
Manipulation of the glutathione system modulates roGFP2 responses 0.5 ppm O_3_ exposure as reported by BEAS-2B cells expressing either roGFP2 or Grx1-roGFP2. (*B*) BEAS-2B cells were pretreated with 100 µM 2-AAPA, a glutaredoxin inhibitor, before exposure to 0.5 ppm O_3_; the responses shown are the normalized 404/488 ratios plotted relative to their established baseline. (*C*) BEAS-2B cells were pretreated with 1 µM sodium selenite for 48 hr before 0.5 ppm O_3_ exposure. To facilitate comparison of the responses in (*A*) and (*C*), the normalized ratios were plotted as a percentage of the signals obtained at maximal oxidizing and reducing conditions achieved using 1 mM H_2_O_2_ and 10 mM DTT, respectively. Other experimental conditions were as described for Figure 2. Values shown are mean ± SE (*n* ≥ 3).

Next, we asked whether the roGFP2 response to O_3_ is influenced by glutaredoxin (Grx) activity. Grx is essential to mediate roGFP2 oxidation by GSSG (Figure 1), but Grx should play no role if roGFP2 is directly oxidized by O_3_. On the one hand, we compared the response of roGFP2 (which interacts with endogenous Grx) with that of Grx1-roGFP2, a translational fusion of Grx1 and roGFP2. The fusion of these components is known to kinetically improve the equilibration between roGFP2 and GSSG in a highly specific manner (Gutscher et al. 2008; Meyer and Dick 2010). We found that the O_3_-induced increase of *E*_GSH_ in BEAS-2B cells expressing Grx1-roGFP2 occurred earlier and at a faster rate relative to that reported by cells expressing unlinked roGFP2, thus indicating a GSSG/Grx–specific response (Figure 4A). In contrast, we investigated the effect of glutaredoxin inhibition by pretreating cells with the dithiocarbamate derivative 2-AAPA (Sadhu et al. 2012). The inhibitor completely blocked roGFP2 responses to O_3_ and H_2_O_2_ (which also act indirectly through glutathione oxidation and reduction, respectively) (Figure 4B), thus confirming the role of Grx as the catalyst necessary for roGFP2 responsiveness to O_3_. Together these observations confirm that O_3_ is not simply sensed by the probe through direct oxidation, but rather by its specific effects on the glutathione redox couple.

Having confirmed that O_3_ induces formation of GSSG, which is then detected by the roGFP2 probe, we asked whether glutathione peroxidases (GPx), major generators of GSSG, are involved in the O_3_ response. Here we investigated the role of GPx activity in O_3_-induced roGFP2 redox changes by pretreating BEAS-2B cells with 1 µM sodium selenite for ≤ 48 hr before O_3_ exposure. Previous studies have used selenium supplementation as an effective means of increasing GPx expression, a finding that we also observed in preliminary studies with BEAS-2B cells [see Supplemental Material, Figure S4 (http://dx.doi.org/10.1289/ehp.1206039)] (Helmy et al. 2000; Holben and Smith 1999; Leist et al. 1996). Selenium-induced overexpression of GPx accelerated roGFP2 oxidation during a 0.5 ppm O_3_ exposure (Figure 4C), suggesting that O_3_ gives rise to peroxides, which are then converted by GPx to GSSG, which is in turn reported by roGFP2 through the intervention of Grx.

**Figure 5:**
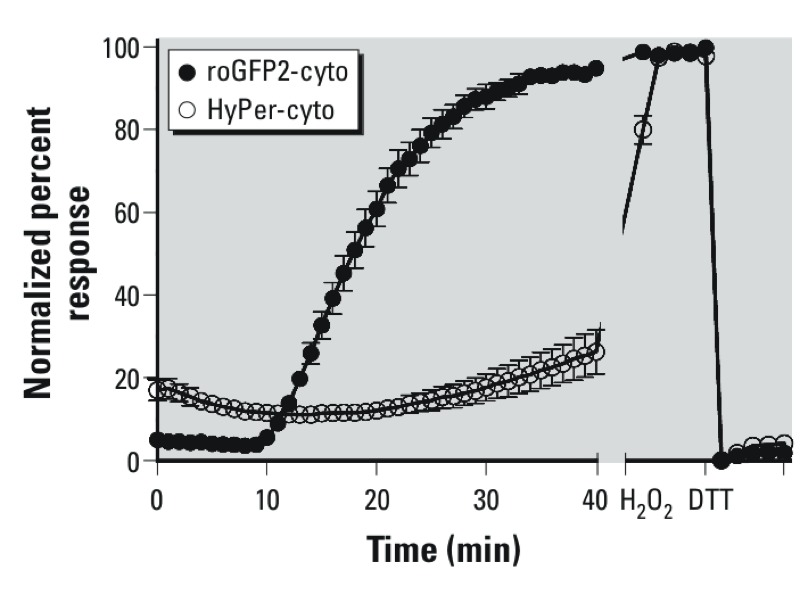
Comparison between roGFP2 and HyPer responses to O_3_. BEAS-2B cells expressing either roGFP2 or the H_2_O_2_ sensor, HyPer, were exposed to 0.5 ppm O_3_ as described for Figure 2. To facilitate comparison, the normalized ratios were plotted as a percentage of the signals at maximal oxidation and reduction achieved using 1 mM H_2_O_2_ and 10 mM DTT, respectively. Values shown are mean ± SE (*n* ≥ 3).

*Investigating the role of secondary products in O_3_-induced redox changes*. Because the data shown in Figure 4C suggested the involvement of peroxides in the O_3_ response, we asked whether there is a specific role for H_2_O_2_. For these experiments we examined H_2_O_2_ generation as a consequence of O_3_ exposure using the cytosolic-targeted H_2_O_2_ sensor, HyPer. O_3_ caused a relatively modest increase in the HyPer response during the exposure period (Figure 5). However, the observed HyPer response did not precede nor match the magnitude of the roGFP2 response, making it unlikely that the observed increase in *E*_GSH_ is primarily caused by H_2_O_2_ generation.

**Figure 6:**
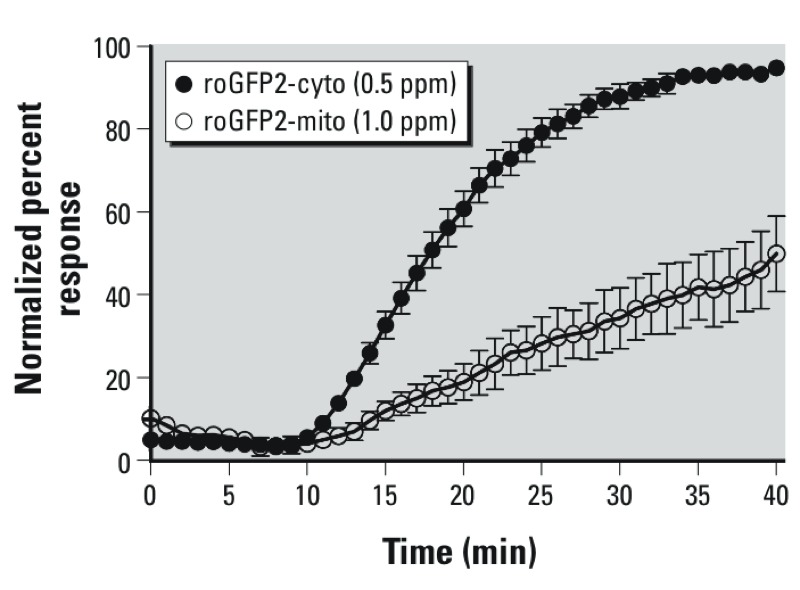
O_3_-induced *E*_GSH_ changes affect the cytosol more rapidly than the mitochondrial matrix. BEAS-2B cells expressing roGFP2 targeted to either the cytosol or mitochondria were exposed to either 0.5 ppm (roGFP2-cyto) or 1.0 ppm (roGFP2-mito). For direct comparison, the normalized ratios were plotted as a percentage of the maximal oxidation achieved using 1 mM H_2_O_2_ and 10 mM DTT. Other experimental conditions were as described in Figure 2. Values shown are mean ± SE (*n* ≥ 3).

Mitochondrial oxidant production, frequently associated with increased oxidation of mitochondrial glutathione, has been implicated as a contributing factor in the cellular response to xenobiotics (Cheng et al. 2010, 2012; Hanson et al. 2004). Therefore, we next used mitochondrially targeted roGFP2 (roGFP2-mito) to assess the impact of O_3_ exposure on the mitochondrial *E*_GSH_ of BEAS-2B cells. As shown in Figure 6, exposure to 1 ppm O_3_, twice the amount used for cytosolic assessments, induced an increase in mitochondrial *E*_GSH_. However, relative to the cytosolic roGFP2 response, the increase in mitochondrial *E*_GSH_ occurred at a slower rate and achieved a lower magnitude, suggesting that mitochondrial oxidants are not the primary source of the oxidants that lead to increased cytosolic *E*_GSH_.

## Discussion

“Oxidative stress” is a frequently cited mechanistic component of the adverse health effects induced by numerous xenobiotic compounds (Bargagli et al. 2009; Chung and Marwick 2010; Ciencewicki et al. 2008; Jones 2008; Kohen and Nyska 2002; MacNee 2001; Ward 2010; Yang and Omaye 2009). However, the term “oxidative stress” is a very broad concept and the detection of early and specific indices of oxidant stress has proven to be methodologically difficult. The advent of genetically encoded fluorescent reporters that are sensitive to their redox environment has enabled real-time imaging-based assessments of oxidant outcomes in living cells with unprecedented spatial and temporal resolution. In this study, we validated the use of one such reporter, roGFP2, for the specific assessment of xenobiotic-induced changes in the *E*_GSH_ using O_3_ as a model toxicant and BEAS-2B cells as a model of the human bronchial epithelium.

The prooxidative change in *E*_GSH_ observed in this study represents an early event in the oxidant injury caused by O_3_. O_3_ is a potent oxidant gas that has the potential to interact directly with virtually any cellular component, potentially including fluorescent reporter molecules such as roGFP2. Thus, in interpreting the probe response observed in O_3_-exposed BEAS-2B cells, we had to consider the possibility that O_3_ could be bypassing the glutathione system through which roGFP2 sensors normally respond (Gutscher et al. 2008; Meyer and Dick 2010).

Our findings strongly suggest that even in the presence of a strong oxidant like O_3_, roGFP2 is oxidized only indirectly through its known coupling to the glutathione system. This conclusion is supported by several observations: First, glucose deprivation increased O_3_-mediated roGFP2 oxidation, consistent with the requirement for NADPH in robustly maintaining *E*_GSH_, the lack of glucose preventing regeneration of reducing equivalents throughout the exposure period. NADPH levels were approximately 70% lower in cells equilibrated in the absence of glucose, which appears to be sufficient to sensitize cells uniformly. In addition, it is important to bear in mind that other cellular processes also draw on the NADPH pool, and the continued lack of glucose largely prevents active regeneration of reducing equivalents throughout the exposure period.

Second, we confirmed the role of glutaredoxin in mediating the roGFP2 response to O_3_. Grx1 is required to transfer oxidative equivalents from the glutathione pool to roGFP2. In previous studies using Grx1-roGFP2, the chimeric linkage of Grx1 to roGFP2 enhanced responses to physiological oxidants such as H_2_O_2_ (Gutscher et al. 2008; Meyer and Dick 2010). Importantly, Grx1-roGFP2 also accelerated the roGFP2 response to O_3_, whereas inhibition of endogenous Grx with 2-AAPA prevented roGFP2 oxidation in the presence of O_3_. Although early reports describe 2-AAPA as being an inhibitor of glutathione reductase, a more recent study reports that this dithiocarbamate derivative acts as a direct inhibitor of Grx as well (Sadhu et al. 2012). Thus, the finding that 2-AAPA–treated cells failed to respond to O_3_ supports the involvement of Grx and is consistent with the claim that 2-AAPA is an inhibitor of Grx. Importantly, the fact that use of this inhibitor was effective at disconnecting the glutathione pool from the redox reporter argues against a nonspecific interaction between O_3_, or a secondary oxidant, and the roGFP2 sensor.

Last, findings from experiments examining the effect of overexpression of glutathione peroxidases (GPx) using prolonged selenite supplementations are consistent with an upstream role of GPx in the oxidative pathway leading to O_3_-induced roGFP2 oxidation. GPx couple the reduction of (hydro)peroxides to the generation of GSSG (Arthur 2000; Matés and Sánchez-Jiménez 1999; Meyer and Dick 2010). In our system, the increased expression of GPx-enhanced roGFP2 oxidation, suggesting that O_3_ exposure generates (hydro)peroxides, which then drive the formation of GSSG. In fact, O_3_ has been shown to produce many types of lipid hydroperoxides upon exposure (Mudway and Kelly 2000; Yang and Omaye 2009). Moreover, cells exposed to O_3_ may have an increased H_2_O_2_ burden as well. Overall, the results from GPx-overexpressing cells suggest that the increased activity of these enzymes leads to an enhanced catalytic destruction of peroxides with concomitant GSH oxidation, leading to the roGFP2 response to O_3_.

Using the H_2_O_2_ probe HyPer, our initial assessments suggested slightly elevated H_2_O_2_ production following O_3_ exposure of BEAS-2B cells. The quantitative interpretation of HyPer responses is, however, difficult. It is not clear to what extent the OxyR domain of HyPer may be outcompeted by endogenous peroxidases. In addition, HyPer is highly pH sensitive, as much as the cpYFP module on which it is based (Schwarzlander et al. 2012). Thus, an O_3_-induced intracellular acidification could dampen the HyPer response to H_2_O_2_. Nevertheless, following O_3_ exposure, HyPer responded to exogenously applied H_2_O_2_ and DTT as expected, which demonstrates the general functionality of the probe. Taken together, the delayed time of onset, the slow rates of response, and the relatively low magnitude of the HyPer responses seem to suggest that H_2_O_2_ production is not a major factor in the total O_3_-induced *E*_GSH_ effects. Likewise, the measurements in mitochondrially targeted roGFP do not support a mitochondrial source for the O_3_-induced increase in cytosolic *E*_GSH_. These results suggest that the relevant oxidant species, potentially a hydroperoxide, is primarily generated in the cytosol or within the outer plasma membrane, and the mitochondrial *E*_GSH_ response would be expected to lag behind that of the cytosol. Additionally, differences in peroxidase composition and activity may contribute to the lag in the O_3_-induced mitochondrial *E*_GSH_ response relative to the cytosol.

The studies presented herein cannot completely exclude a partial contribution of direct interactions between O_3,_ or its secondary by-products, with the thiols of the roGFP2 sensor. In addition, because the roGFP2 fluorescence ratio reflects *E*_GSH,_ which is a function of both the GSSG:GSH ratio and the total glutathione concentration, it is possible that O_3_-induced electrophilic attack mediates the changes reported by roGFP2 by consuming reduced GSH. It should, however, be noted that the *E*_GSH_ in the cytosol or mitochondrial matrix is much more sensitive to an increase in GSSG than to depletion of GSH. This is because the *E*_GSH_ in the cytosol (around –320 mV, or even lower, in mitochondria) represents nanomolar GSSG in a millimolar pool of GSH. To deflect the roGFP2 signal from –320 mV to about –260 mV only requires the concentration of GSSG to increase from 200 nm to 20 µM (in a 10 mM total glutathione pool). To achieve the same magnitude of *E*_GSH_ response by depleting GSH exclusively would require a loss of 90% of GSH (e.g., from 10 mM to 1 mM) (Meyer and Dick 2010). Such a massive depletion of GSH by parts-per-million concentrations of O_3_ (which could generate only limited amounts of electrophiles) seems stochiometrically unlikely. If one also considers the relative kinetics of GSSG generation and consumption (GSSG generation by GPx exhibits second order rate constants in the range of 1 × 10^8^ M^–1^ sec^–1^), it appears reasonable to suggest that the O_3_ effect reported in our study is primarily due to GSSG generation and that the contribution of glutathionylated electrophile(s) formation is minor.

Thus, although several different O_3_-induced processes may together drive glutathione oxidation, including lipid peroxidation, H_2_O_2_ generation, and other oxidizing processes, the available evidence strongly suggests that the cytosolic roGFP2 responses to O_3_ exposure are appropriately reporting the *E*_GSH_. Taken together, these results demonstrate that roGFP2-based sensors can be used to monitor shifts in glutathione redox homeostasis in O_3_-exposed cells. Furthermore, the experimental approach we used may be utilized for the validation of “oxidant stress” induced by other reactive xenobiotics in living cells.

## Conclusion

Our results demonstrate the utility of using genetically encoded fluorescent reporters in making reliable assessments of cells undergoing exposure to xenobiotics with strong oxidizing properties.

## Supplemental Material

(1.1 MB) PDF
