# Supplementary material for: Monitoring Intracellular Redox Changes in Ozone-Exposed Airway Epithelial Cells
Source: Environ Health Perspect. 2012 Dec 18;121(3):312–7. doi: 10.1289/ehp.1206039 (PMC3621206; doi:10.1289/ehp.1206039)
Supplement: (1.1 MB) PDF [file ehp.1206039.s001.pdf]

Supplemental Material

Monitoring Intracellular Redox Changes in Ozone-Exposed Airway Epithelial Cells

Eugene A. Gibbs-Flournoy, Steven O. Simmons, Philip A. Bromberg, Tobias P. Dick, and James M. Samet

Table of Contents:

Figure S1. Stage-top ozone exposure system ....Pg. 2

Figure S2. Determination of NADPH levels ....Pg. 3

Figure S3. Determination of intracellular glutathione ....Pg. 4

Figure S4. Selenium-induced GPx1 overexpression ....Pg. 5

Figure S5. Dose- and time-dependent increase in E_{GSH} with cell images....................Pg. 6

Supplemental Figure S1

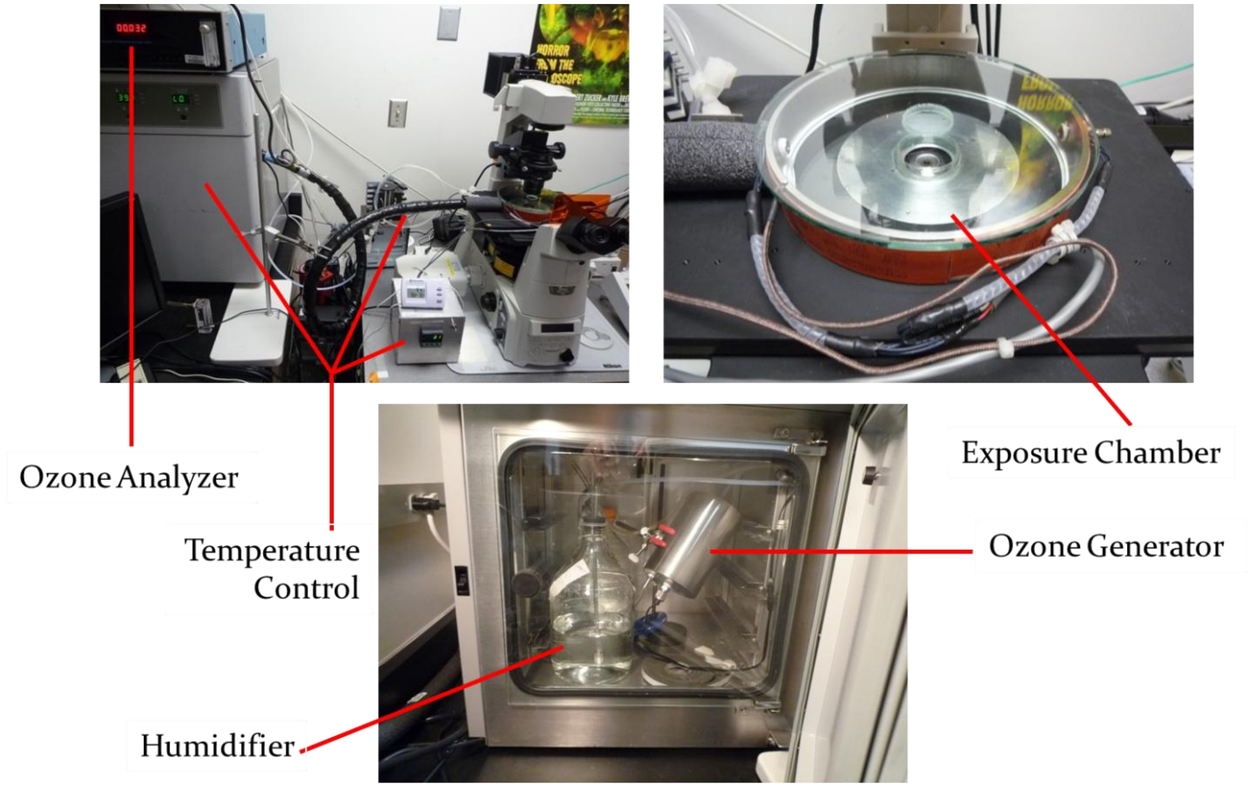

Figure S1. Stage-top ozone exposure system. Displayed are components of the stage-top ozone (O_3) exposure system used for all real-time assessments of living cells undergoing O_3 exposure. 5% CO_2/Balance Air entering the system is first humidified by bubbling it through autoclaved dH_2O housed in a 37°C incubator. The gas is then passed through an O_3 generator where O_3 is created by using a UV pen lamp. Concentrations of O_3 are controlled by a voltage regulator which adjusts lamp intensity. The newly generated O_3 then flows to a temperature-controlled, stage-top exposure chamber via a heated line. All gas entering the exposure chamber is sampled by a Dasibi ozone analyzer at a T-fitting placed at the end of the heated line, just before the entrance to the chamber. The humidity of gas diverted to the O_3 analyzer is removed using a Perma Pure gas drier. All heated components are maintained at or above 37°C to maintain ≥90% humidity of the flowing gas.

Supplemental Figure S2

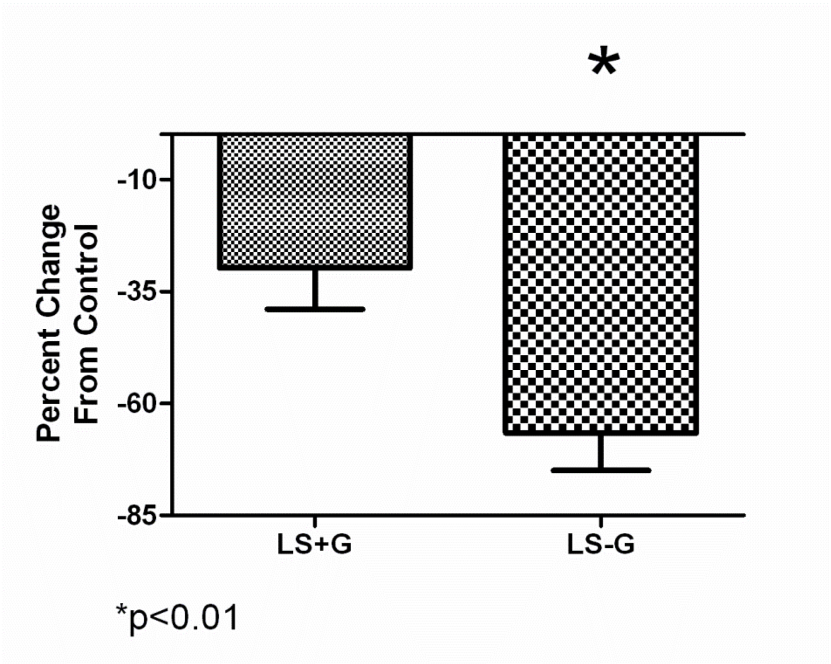

Figure S2. Determination of NADPH levels. The effect of glucose deprivation on NADPH was assessed following a 2 hr equilibration in either Locke Solution with glucose (LS+G) or Locke Solution without glucose (LS-G), and expressed as percent change from the mean NADPH concentration measured from cells maintained in growth media (KGM). *$p<0.01$, $n\geq3$ or more experiments.

Supplemental Figure S3

A

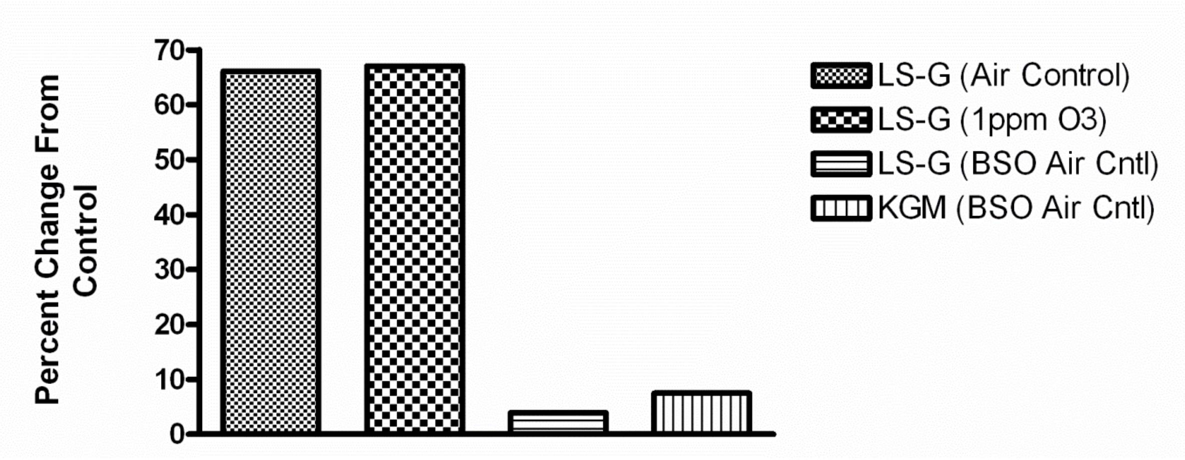

B

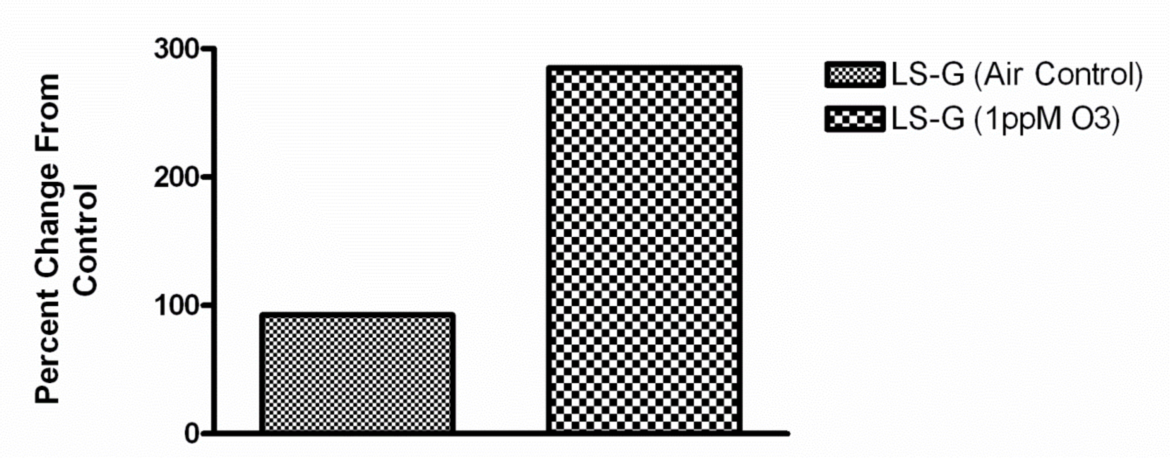

Figure S3. Determination of intracellular glutathione. A) The total glutathione content (GSH + GSSG) of cells exposed to either 0 (air control) or 1 ppm O_3 in Locke solution without glucose (LS-G) for 50 min. To ensure the total glutathione content could be modulated, cells were exposed to an air control in either LS-G or growth medium (KGM) following pretreatment with buthionine sulfoximine (BSO), a gamma-glutamylcysteine synthetase inhibitor used to decrease gluthatione levels. The responses are plotted as percent change from the KGM air control. **B)** The oxidized glutathione content (GSSG) of cells exposed either 0 (air control) or 1 ppm O_3 for 50 min in LS-G. Cells exposed to 0 ppm O_3 in growth media (KGM) serve as the control, and responses are plotted as percent change from the control.

Supplemental Figure S4

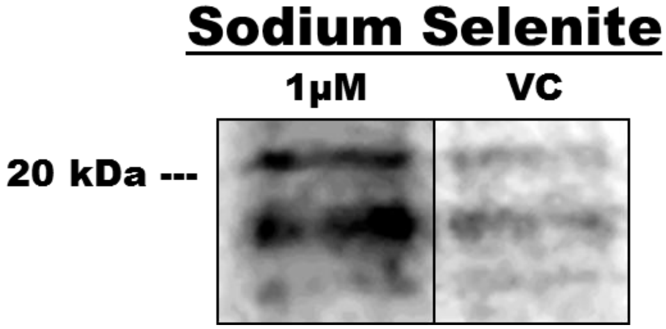

Figure S4. Selenium-induced GPx1 overexpression. Detection of GPx1 expression following a 48 hr pretreatment with either 0 (vehicle control, VC) or 1 uM sodium selenite; cell lysates were separated by SDS-PAGE and immunoblotted with anti-GPx1. The expected size of GPx1 is approximately 22 kDa.

Supplemental Figure S5

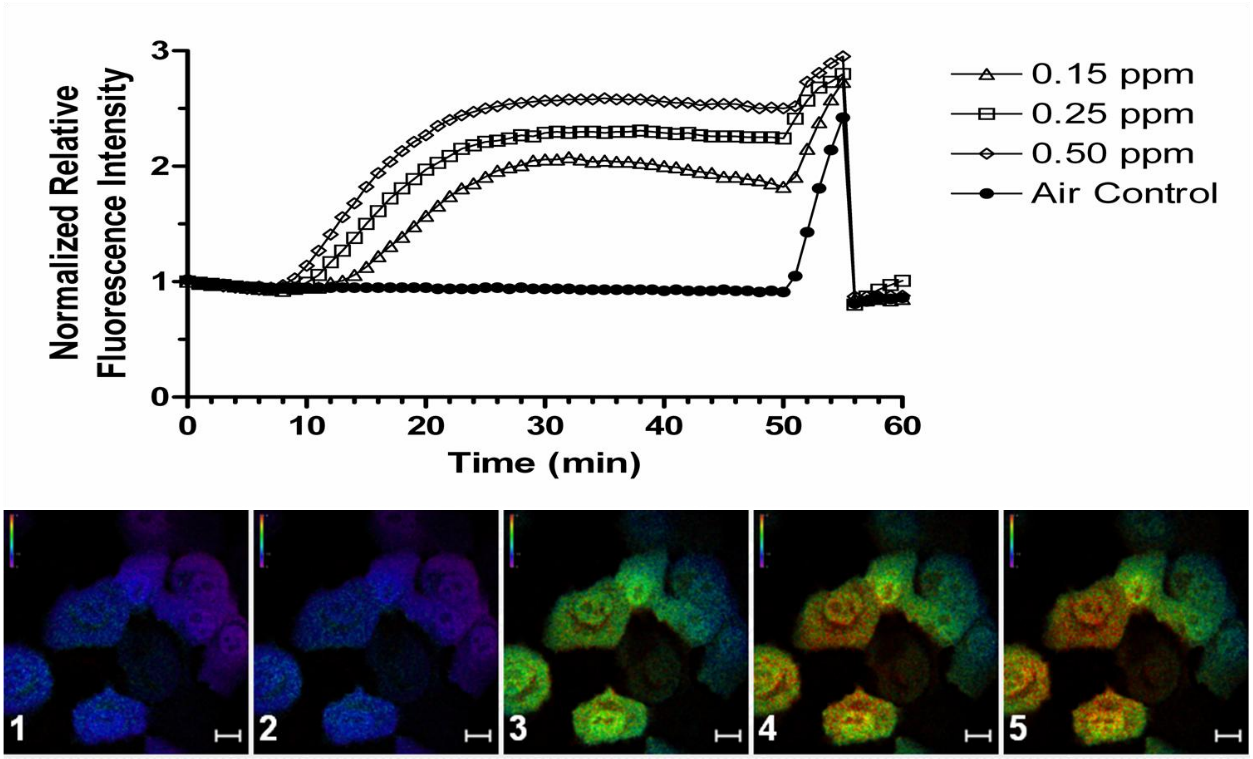

Figure S5. Exposure to O_3 induces a dose- and time-dependent increase in the cytosolic glutathione redox potential in airway epithelial cells. BEAS-2B cells expressing cytosolic roGFP2 were exposed to clean air for 5 min followed by a 0 (Air Control), 0.15, 0.25 or 0.50 ppm O_3 exposure for 35 min in a stage-top exposure system maintained at 37 ^{o}C, >90% relative humidity, and 5% CO_2. Shown are the ratiometric values (404/488) calculated from the fluorescence intensity emitted at 510 nm induced by sequential excitation at 404 and 488 nm, and plotted relative to the 5 min baseline. Addition of 0.1 mM H_2O_2 at the end of the O_3 exposure produced a maximal response which was fully reversible with the addition of 10 mM DTT. The pseudo-colored images displayed below the plotted data represent ratiometric changes in fluorescence of cells being exposed to 0.5 ppm O_3. Increases in the ratiometric fluorescence intensity are represented by changes in color from Violet/Blue to Red as indicated by the scale bar in the upper left hand corner. Each panel was taken in series at 10 min intervals starting at T=0 min for panel 1 and ending with T=40 min for panel 5. The scale bar in the bottom right corner of each panel is 10 µm in length. The data shown were derived from 3 or more separate experiments monitoring 7 or more cells in real-time throughout the exposure period.
